# Understanding Mental Health App Use Among Community College Students: Web-Based Survey Study

**DOI:** 10.2196/27745

**Published:** 2021-09-14

**Authors:** Judith Borghouts, Elizabeth V Eikey, Gloria Mark, Cinthia De Leon, Stephen M Schueller, Margaret Schneider, Nicole Stadnick, Kai Zheng, Dana B Mukamel, Dara H Sorkin

**Affiliations:** 1 Department of Medicine University of California Irvine Irvine, CA United States; 2 Herbert Wertheim School of Public Health and Human Longevity Science University of California, San Diego San Diego, CA United States; 3 The Design Lab University of California, San Diego San Diego, CA United States; 4 Department of Informatics University of California, Irvine Irvine, CA United States; 5 Department of Psychological Science University of California, Irvine Irvine, CA United States; 6 Department of Public Health University of California, Irvine Irvine, CA United States; 7 Department of Psychiatry University of California, San Diego La Jolla, CA United States; 8 Dissemination and Implementation Science Center UC San Diego Altman Clinical and Translational Research Institute La Jolla, CA United States; 9 Child and Adolescent Services Research Center San Diego, CA United States

**Keywords:** mHealth, mental health, community college, students, structural equation modeling, mobile apps, services, mental health services, stress, privacy

## Abstract

**Background:**

Mental health concerns are a significant issue among community college students, who often have less access to resources than traditional university college students. Mobile apps have the potential to increase access to mental health care, but there has been little research investigating factors associated with mental health app use within the community college population.

**Objective:**

This study aimed to understand facilitators of and barriers to mental health app use among community college students.

**Methods:**

A web-based survey was administered to a randomly selected sample of 500 community college students from April 16 to June 30, 2020. Structural equation modeling was used to test the relationships between the use of mental health apps, perceived stress, perceived need to seek help for mental health concerns, perceived stigma, past use of professional mental health services, privacy concerns, and social influence of other people in using mental health apps.

**Results:**

Of the 500 participants, 106 (21.2%) reported use of mental health apps. Perceived stress, perceived need to seek help, past use of professional services, and social influence were positively associated with mental health app use. Furthermore, the effect of stress was mediated by a perceived need to seek help. Privacy concerns were negatively associated with mental health app use. Stigma, age, and gender did not have a statistically significant effect.

**Conclusions:**

These findings can inform development of new digital interventions and appropriate outreach strategies to engage community college students in using mental health apps.

## Introduction

### Background

Mental health concerns are a significant issue among college students [1,2], and the last decade has seen a rise in mental health concerns among students [3]. Community college students, in particular, face a growing crisis of mental health concerns. A survey conducted by the Wisconsin HOPE Lab found that almost 49.4% of respondents across 10 community colleges in seven states reported mental health issues [4]. A survey among 39,930 community college students found that 60% of respondents were housing insecure in the previous year, and 19% of respondents had been homeless [5]. These basic needs insecurities not only impact students’ performance at school but also have been associated with poorer physical and mental health [6,7].

College students can face multiple barriers to accessing support and resources, and these barriers may be more pronounced for students enrolled in community college. For example, the community college population has a higher proportion of students who are single parents and/or who are working jobs while attending classes, and the students are more likely to come from a lower socioeconomic background compared to university students [8]. Whereas high workload and academic stress are common issues raised among university students, financial or housing stress may be more common among community college students [9,10]. Furthermore, community college students have less access to mental health services than university students, and they may be in particular need of increased access to mental health resources [9]. Students may also not recognize a need to seek help, or they may feel uncomfortable discussing mental health problems [11].

Given college students’ limited access to mental health resources, studies have explored the use of technology-based solutions, such as mobile apps [12-14]. Mobile apps for health purposes are one of the fastest growing categories of apps, and currently, more than 10,000 mental health apps are publicly available [15]. Mental health apps may be particularly useful for students: according to a Pew Research 2019 study on mobile phone ownership, 85% of people attending college own a smartphone, and 96% of people aged 18-29 years own a smartphone [16]. Furthermore, students and young adults are active users of the internet for seeking health information [2,10,17,18].

To inform development and deployment of mental health apps for community college students, an understanding of students’ mental health needs and current use of apps is necessary. However, community college students are often underrepresented in the discussion of student mental health [8,9], and the mechanisms behind community college students’ use of mental health apps are not yet fully understood, despite the fact that there are 11.8 million students enrolled in community colleges in the United States [19]. There are differences in university and community college environments with regards to demographics, culture, and mental health issues [9,10]. These differences between community college and university student populations may affect students’ mental health needs and subsequent app use. To address this gap, this paper aimed to address what factors are associated with community college students’ use of mental health apps. Specifically, our study focused on 6 factors that prior research has found to affect use of mental health resources: perceived need to seek help for mental health concerns, perceived stigma, past use of professional mental health services, privacy concerns, and social influence of other people in using mental health apps.

### Mental Health App Interest and Use Among Students

With the growing development and ubiquity of mental health apps, research has been performed on both interest in and use of these apps among students. Kern et al [20] conducted a needs assessment among 741 university students about their attitude toward mental health apps. The researchers found that there is interest in mental health apps among students, especially among students who had received professional mental health services in the past 12 months. The primary reasons for interest in mental health apps were confidentiality, convenience, and immediate availability. However, despite interest, actual use was limited, highlighting the importance to understand what factors are associated with adoption and app use.

### Perceived Stress

Experiencing stress can be a starting point for students to adopt mental health apps. Perceived stress has been linked with interest in, and acceptance of, stress management apps [21,22]. This link indicates that people with an actual need for support are more likely to accept and use mental health interventions.

### Help-Seeking Behavior Among Students

For a person to use a health intervention, there must be a motivation and willingness to use it [23]. The Health Belief Model [23] explains the adoption of health interventions through several constructs related to an individual’s beliefs about their condition. It argues that a person’s belief in the severity of their illness or health symptoms, and the perceived benefits of seeking treatment for these symptoms, affect their adoption of health interventions. These constructs suggest that the mere presence of symptoms is insufficient: the individual also has to believe that there is benefit in seeking help for these symptoms.

### Stigma

Previous studies found that a common barrier among young people and students in seeking traditional mental health resources is stigma associated with mental health [11,24]. A survey study with university students found that, despite overall poor mental well-being across the study sample, there was only a weak relationship between mental well-being and use of mental health resources, and this relationship was mediated by stigma [25]. In other words, if students are in need of help but experience a high level of stigma, that stigma may still prevent them from seeking resources such as apps.

It is argued that services delivered through technology can overcome the barrier of stigma because people can use the technology privately, and other people do not have to know they are seeking help [20]. However, it has not been evaluated if and how perceived stigma is associated with use of apps and how it may mediate the relationship between stress and mental health app use.

### Past Use of Professional Services

Past use of mental health services has also been associated with app use. A person’s prior use of mental health services can increase their interest in using mental health technologies [20]. A positive experience with these services can increase the likelihood that people will be open to trying a digital intervention [26-28].

### Privacy

An important aspect of mental health apps among university students is confidentiality. Students in some studies thought that mental health apps can facilitate confidentiality [20], and they did not think that privacy would be an issue as long as their data were anonymous [29]. University students in a focus group study, however, expressed concerns that their data would leak and that people such as their lecturers or tutors could access their data [14]. It is therefore expected that privacy concerns can be a challenge or barrier to mental health apps and will negatively affect app use.

### Social Influence

Expectations and beliefs about mental health apps by other people close to the user, such as family, friends, or health providers, may also play a role in mental health app use. The Technology Acceptance Model (TAM) [30] includes the importance placed on other people’s opinions, labelled social influence, as a predictor of technology adoption. Including social influence as a component of technology adoption is based on the theory of reasoned action (TRA), in which user actions are a result of the user’s perception that significant others expect them to conduct these actions [31].

In general, although previous studies have identified what students perceive to be important aspects of mental health apps, it is less understood how these aspects are associated with use. Moreover, most studies included university students, but community college students can experience different mental health issues and barriers to accessing resources, which can affect app use.

Our study aimed to understand how perceived stress, perceived need to seek help, perceived stigma, past use of professional mental health services, privacy concerns, and social influence are associated with mental health app use among community college students. Our findings can be used to guide development and selection of digital mental health interventions that address community college students’ needs, and they can inform effective outreach and engagement strategies to engage community college students with these interventions.

### Hypotheses

Based on prior studies, the hypotheses of the study are:

1. Perceived stress is positively associated with students’ use of mental health apps.
2. The association of perceived stress and use of mental health apps is mediated by a perceived need for help.
3. The association of perceived stress and use of mental health apps is mediated by perceived stigma.
4. Past use of professional mental health services is positively associated with students’ use of mental health apps.
5. Privacy concerns are negatively associated with mental health app use.
6. Social influence is positively associated with students’ use of mental health apps.

## Methods

### Study Design

A random sample of 5000 students at a community college in California were selected to participate in this study. To obtain a sample that was balanced for gender and race, sampling was in proportion to the demographics (gender/race) of California community colleges [32]. The demographics for California community colleges are as follows: 26% identify as White, 45% as Hispanic/Latinx, 12% as Asian, and 6% as African American. Approximately half of community college students (54%) identify as female.

Students’ email addresses were obtained through the College’s registrar’s office. These addresses were provided to a staff member at the College, who distributed the invitational emails. The web-based survey took 20-30 minutes to complete. Participants received a US $10 gift card for completing the survey.

### Participants

The email was received by 4985 students (15 emails bounced back). A total of 574 participants started the survey, resulting in a response rate of 11.5% (574/4985). A total of 500 participants completed the survey. The survey participants had a mean age of 23.8 years (SD 8.0), and 314/500 (62.8%) identified as female. Of the 500 students, 137 (27.4%) identified as White, 119 (23.8%) identified as Hispanic/Latinx, 66 (13.2%) identified as Asian, and 32 (6.4%) identified as African American. With the exception of the proportion of Hispanic/Latinx participants, our study sample broadly matches the breakdown of demographics for California community colleges.

Most reported their primary language as English (310/500, 62%), while 27% (135/500) reported Spanish as their primary language; the survey was only available in English. Of the 500 participants, 87 (17.4%) had an annual household income of less than US $10,000. The majority of participants (402/500, 80.4%) had health insurance, but only 23.9% (96/402) of those with insurance were sure that their plan provided coverage for mental health services. Further demographic characteristics are shown in Table 1.

**Table 1:** Demographics of the study participants (N=500).

Demographics		Value^a^	
Age (years), mean (SD)		23.8 (8)	
**Gender^b^, n (%)**			
	Male	171 (34.2)	
	Female	314 (62.8)	
**Sexual orientation^c^, n (%)**			
	Heterosexual or straight	391 (78.2)	
	Bisexual	39 (7.8)	
	Questioning or unsure of sexual orientation	18 (3.6)	
**Enrollment status, n (%)**			
	Full-time	306 (61.2)	
	Part-time	167 (33.4)	
**Time of classes, n (%)**			
	Daytime	377 (75.4)	
	Evening	83 (16.6)	
**Employment status, n (%)**			
	Unemployed	213 (42.6)	
	Part-time	158 (31.6)	
	Full-time	63 (12.6)	
	Retired	5 (1)	
**Race, n (%)**			
	White	137 (27.4)	
	Hispanic/Latino/a/x	119 (23.8)	
	Asian	66 (13.2)	
	More than one race	44 (8.8)	
	Black or African American	32 (6.4)	
	American Indian or Alaska Native	3 (0.6)	
	Native Hawaiian or other Pacific Islander	3 (0.6)	
**Ethnicity, n (%)**			
	Mexican/Mexican-American/Chicano	183 (36.6)	
	More than one ethnicity	55 (11)	
	Asian	52 (10.4)	
	European	46 (9.2)	
	Central American	37 (7.4)	
	African	18 (3.6)	
	Middle Eastern	13 (2.6)	
	South American	12 (2.4)	
	Eastern European	10 (2)	
**Primary language, n (%)**			
	English	310 (62)	
	Spanish	135 (27)	
	Vietnamese	7 (1.4)	
	Arabic	6 (1.2)	
	Mandarin	4 (0.8)	
	Russian	3 (0.6)	
**Marital status, n (%)**			
	Single	289 (57.8)	
	In a committed relationship	146 (29.2)	
	Married	35 (7)	
	Divorced or separated	9 (1.8)	
**Children, n (%)**			
	Yes	69 (13.8)	
	No	416 (83.2)	
**Living situation, n (%)**			
	Live with family	389 (77.8)	
	Live with spouse or partner	45 (9)	
	Live alone	18 (3.6)	
	Live with roommate(s)	14 (2.8)	
	Live with children	10 (2)	
**Homeless, n (%)**			
	Yes	12 (2.4)	
	No	472 (94.4)	
**Household income (US $), n (%)**			
	<10,000	87 (17.4)	
	10,0000-29,999	131 (26.2)	
	30,000-49,999	59 (11.8)	
	50,000-89,999	52 (10.4)	
	90,000 or above	49 (9.8)	
**Disability**			
	Yes	47 (9.4)	
	No	426 (85.2)	
**Veteran**			
	Yes	8 (1.6)	
	No	480 (96)	
**Health insurance**			
	Yes	402 (80.4)	
	No	61 (12.2)	

^a^Not all respondents answered each question; hence, some percentages do not sum to 100%.

^b^Multiple genders were included as options on the survey; however, participants reported their genders as only male or female.

^c^Other sexual orientations were included as options on the survey; however, the results in the table reflect the participants’ responses.

### Measures

The complete survey instrument is included in Multimedia Appendix 1.

#### Barriers to Mental Health Resources, Important Aspects of Mental Health Apps, and Activities People Would Like to Do Using Mental Health Apps

Participants were asked to report on barriers they faced to accessing mental health–related resources, important aspects about using mental health apps, and what they would like to do using mental health apps. For each of these questions, they were instructed to “select all that apply” from a list of options and/or give an answer in their own words. The list of barrier options was taken from the Healthy Minds Study, an annual web-based survey assessing mental health and service use among students [3].

#### COVID-19

Survey responses were collected between April 16 and June 30, 2020, during the COVID-19 pandemic. Participants were asked questions related to whether and how COVID-19 had impacted their lives, to gauge changes resulting from the pandemic. These results were not used in the analysis but are reported here to characterize the impact of COVID-19 on the participants. Of the 500 students, 107 (21.4%) knew someone who had been diagnosed with COVID-19. Of these 107 participants, 77 (72.0%) reported that the person diagnosed was a friend or acquaintance; for 37 (34.6%) it was a family member; for 11 (10.3%) it was a colleague; and 4 (3.7%) said they had been diagnosed themselves.

#### Mental Health

A single dichotomous (yes or no) item asked participants whether they were currently experiencing or had ever experienced a mental illness. In the survey, it was explained that while the term mental illness was used, there are many different terms that can be used, such as mental health problem, emotional distress, psychological disorder, and mental challenge.

#### Mental Health App Use

A single question was used to identify whether participants had used mental health apps. In this study, a mental health app was defined as “an application on your mobile phone or tablet device that helps you manage your mental, emotional, or psychological health or get access to resources to support your mental, emotional, or psychological health.” Participants could respond by indicating whether they had used apps in the past, were currently using apps, had never used apps but would be interested in doing so, or had never used apps and were not interested in doing so. In this paper, those who had used or were currently using mental health apps were defined as users, and those who had never used mental health apps were defined as nonusers.

#### Past Use of Professional Mental Health Services

Two dichotomous (yes or no) response items from the California Health Interview Survey [33] were used to identify whether participants had sought help from their general practitioner or another professional, such as a counselor, for mental health concerns in the past 12 months.

#### Perceived Stress

The 7-item version of the College Student Stress Scale [34] was used to assess perceived stress. A higher score on this scale indicates a higher level of stress. The validity and reliability of this scale have been validated with college students (α=.87) [34].

#### Perceived Need to Seek Help

A dichotomous (yes or no) response item from the California Health Interview Survey [33] was used to identify whether participants felt they may have needed to see a professional because of problems with their mental health in the past 12 months.

#### Mental Health Concerns

Participants who answered they felt a need to see a professional in the past 12 months were asked to select what mental health concerns, if any, they experienced in these past 12 months. Participants could give an answer in their own words and/or select all options that applied from a list. Examples of mental health concerns were stress, depression, anxiety, loneliness, and concerns related to interpersonal relationships.

#### Perceived Stigma

The Perceived Stigma subscale of the Depression Stigma Scale [35] was used to assess the perceived stigma participants experience toward mental health. There are multiple types of stigma, and we chose to include this particular type because it can be measured for all participants, regardless of whether they have experienced a mental illness. Participants were asked to rate 9 statements related to perceived stigma (eg, “Most people believe that people with a mental illness could snap out of it if they wanted”), using a Likert scale from 1, strongly disagree, to 4, strongly agree. A higher score indicates greater perceived stigma. Internal consistency of reliability of the Perceived Stigma scale has been validated in prior work (Cronbach α=.82) [35].

#### Social Influence

Social influence was measured using three statements (eg, “People who are important to me think I should use mental health apps”) based on the Unified Theory of Acceptance and Use of Technology (UTAUT) questionnaire [36], which is used to evaluate people’s technology acceptance and adoption. Participants were asked to rate these statements using a Likert scale from 1, strongly disagree, to 5, strongly agree. A higher score indicates greater importance placed on other people’s expectations.

#### Privacy Concerns

Privacy concerns were measured using 6 statements (eg, “I feel that as a result of my using mental health apps, others know more about me than I am comfortable with”) based on the Scale on Mobile Users’ Information Privacy Concerns [37]. The original items were adapted to refer to mental health apps specifically (eg, an original item was “I feel that as a result of my using mobile apps, others know more about me than I am comfortable with”). Participants were asked to rate these statements using a Likert scale from 1, strongly disagree, to 5, strongly agree. A higher score indicates greater concerns. The items were worded slightly differently for participants who answered they had not used mental health apps compared to participants who had used such apps (eg, “I feel that if I were to use mental health apps, others know more about me than I am comfortable with” vs “I feel that as a result of my using mental health apps, others know more about me than I am comfortable with”).

### Factor Analysis and Checking for Multicollinearity

The structural model included two latent variables: privacy and social influence. These were included as latent variables because there were multiple survey items related to the concepts of privacy and social influence, and these were adapted from validated scales to refer to mental health apps specifically. Use of the total score of these adapted versions has not been tested in prior work. Combining the related items into a latent variable, rather than treating them as separate variables, reduces the dimensionality of the data.

The variables of perceived stress and perceived stigma were included in the structural model as observed variables. The reliability of using the total score of these scales been tested and validated in prior work, and they have each been used as one aggregated continuous score in previous models [38]. Aggregating these scales as observed variables, rather than latent variables, was therefore deemed appropriate, and this approach makes it easier to interpret the data.

The latent variables were first assessed using confirmatory factor analysis. Two indices were used to assess the fit of the measurement models: the comparative fit index (CFI) and Tucker-Lewis index (TLI) (the threshold value of acceptable model fit for the CFI and TLI is at least 0.90; see [39]). Maximum likelihood was used as an estimator.

Privacy was measured in the survey with 6 questions (see Multimedia Appendix 1). A test of the fit of an initial model including all 6 privacy questions showed incremental fit indices that were below the acceptable threshold of 0.90 (χ^2^_21_=326.712, CFI=0.85, TLI=0.75). A correlation matrix (see Table 2) identified two groups of correlated items, with intercorrelation values >0.7. The privacy construct was therefore split into two separate privacy constructs: one construct related to information being visible by others (Privacy Construct 1) and one construct related to how information is used (Privacy Construct 2). Two measurement models, one with the first 3 privacy questions and one with the remaining 3 privacy questions, did show an acceptable model fit (χ^2^_6_=162.443, CFI=1.0, TLI=1.0, and χ^2^_6_=147.822, CFI=1.0, TLI=1.0, respectively). The Cronbach α values for the two scales were .91, indicating good internal consistency.

The social influence construct comprised 3 questions (see Multimedia Appendix 1). Confirmatory factor analysis for the social influence construct also showed an acceptable model fit (χ^2^_6_=1155.902, CFI=1.0, TLI=1.0). The Cronbach α value for the scale was .95, showing good internal consistency. The questions were therefore deemed suitable to combine into a latent variable for the structural model.

Before the structural model was tested, the variance inflation factors of the model variables were assessed to detect potential multicollinearity. The variance inflation factors of the variables were all under 1.3, indicating no problematic multicollinearity. The correlation matrix of the variables is displayed in Table 3. The correlation values further show that there were no strong correlations (correlation values exceeding 0.70, see [40]) among the constructs.

**Table 2:** The correlation matrix of privacy items showing two correlated groups of items (Group 1, consisting of items 1, 2 and 3, and Group 2, consisting of items 4, 5, and 6).

	Privacy_1	Privacy_2	Privacy_3	Privacy_4	Privacy_5	Privacy_6
Privacy_1	1					
Privacy_2	0.70	1				
Privacy_3	0.73	0.74	1			
Privacy_4	0.44	0.56	0.61	1		
Privacy_5	0.47	0.56	0.61	0.86	1	
Privacy_6	0.43	0.56	0.62	0.87	0.88	1

**Table 3:** Correlation matrix of model variables showing no strong correlations between variables.

	Perceived stress	Perceived need to seek hep	Past use of professional mental health services	Stigma	Social influence	Privacy Construct 1	Privacy Construct 2	Age	Gender
Perceived stress	1								
Perceived need to seek help	0.40	1							
Past use of professional mental health services	0.06	0.33	1						
Stigma	0.23	0.20	0.11	1					
Social influence	0.08	0.24	0.09	0.08	1				
Privacy Construct 1	0.17	0.09	0.01	0.21	0.20	1			
Privacy Construct 2	0.17	0.12	–0.01	0.16	0.06	0.64	1		
Age	–0.07	–0.09	–0.03	0.01	–0.05	0.00	0.05	1	
Gender	0.21	0.28	0.11	0.10	0.12	0.05	0.07	0.03	1

### Analysis

The dependent variable of the model was the participants’ mental health app use as a dichotomous (yes or no) variable. The independent variables were perceived stress, perceived need to seek help (shortened in the *Results* section as perceived need), past use of professional mental health services (shortened in the *Results* section as past use of services), perceived stigma, social influence, and privacy concerns. Age and gender were added as covariables.

To address Hypotheses 1, 4, 5, and 6, the model tests the effects of stress, past use of services, privacy concerns, and social influence on app use. To address Hypotheses 3 and 4, the model tests whether the effect of stress on app use is mediated by perceived need and perceived stigma.

The relationships between measured variables were tested using structural equation modeling (SEM). SEM was used because it allows the flexibility to include privacy concerns and social influence, which were composed of a subselection and/or adapted items from validated scales as unobservable latent variables, and it allows testing for mediation effects of perceived need and perceived stigma [41]. Latent variables are unobserved constructs that are measured by a number of observed variables (ie, items).

Two indices were used to assess the fit of the structural model: the standardized root mean square residual (SRMR) and root mean square error of approximation (RMSEA) (a SRMR value of <0.08 or an RMSEA value of <0.06 indicates that the model fits the data well; see [39]). Maximum likelihood was used as estimator for the structural equation models. Bootstrapping was used to examine the significance of indirect mediating effects [42]. We used bias-corrected bootstrapping with 1000 samples.

We compared the fit of the full mediation model, which tests for mediation effects of perceived need and perceived stigma, with a direct effect model. The direct effect model tested the direct effects of the independent variables on the dependent variable without considering mediating effects.

In this study, the focus was on testing the mediating effect of stigma on app use. It is imaginable that stigma is related to other independent variables—for example, stigma may be related to higher privacy concerns, or a high level of stigma may be negatively related to past use of services. To understand these interrelationships, intercorrelations among independent variables were determined before the structural model was tested.

Full maximum likelihood was used to impute missing data on scales (ie, perceived stress, perceived stigma, social influence, and privacy concerns). Participants with missing data on dichotomous model variables (ie, perceived need and past use of services) were excluded from the model. The software environment R (R Foundation for Statistical Computing) was used for statistical analysis, and the R package lavaan was used for the structural equation models, bootstrapping, and confirmatory factor analysis [43].

## Results

### Demographic Information

Of the 500 participants, 189 (37.8%) reported that they had experienced a mental illness; 219 (43.8%) of participants reported mental health concerns related to stress. Over half of the participants (262/500, 52.4%) had a stigma score of 21 or higher, suggesting moderate to severe perceived stigma. Of the 500 participants, 106 (21.2%) had used a mental health app. Other descriptive statistics are summarized in Table 4.

**Table 4:** Overview of responses to survey items (N=500).

Variable			Value
**Technology ownership, n (%)**			
	Smartphone	443 (88.6)	
	Desktop or laptop computer	463 (92.6)	
	Tablet	138 (27.6)	
	Mobile/cell phone but not a smartphone	32 (6.4)	
**Technology use, n (%)**			
	Access to Wi-Fi	448 (89.6)	
	Access to a mobile data plan	440 (88)	
	Use of internet constantly or many times per day	452 (90.4)	
**Mental health app use, n (%)**			
	Current user	34 (6.8)	
	Past user	72 (14.4)	
	Nonuser, interested in using apps	199 (39.8)	
	Nonuser, not interested in using apps	180 (36)	
**Mental illness (self-reported), n (%)**			
	Yes	189 (37.8)	
	No	260 (52)	
	Prefer not to answer	51 (10.2)	
**Most common mental health concerns, n (%)**			
	Stress	219 (43.8)	
	Anxiety	207 (41.4)	
	Depression	172 (34.4)	
**Use of professional services in the past 12 months, n (%)**			
	Yes	115 (23)	
	No	374 (74.8)	
**Perceived need to seek help, n (%)**			
	Yes	221 (44.2)	
	No	221 (44.2)	
	Prefer not to answer	21 (4.2)	
**Most common barriers to accessing mental health resources, n (%)**			
	I prefer to deal with issues on my own	260 (52)	
	Financial reasons (eg, too expensive)	141 (28.2)	
	I am concerned about privacy	129 (25.8)	
	I question how serious my needs are	126 (25.2)	
	I worry what other people will think of me	116 (23.2)	
**Most important aspects of using mental health apps, n (%)**			
	The app is free	429 (85.8)	
	Personal information will be kept private	397 (79.4)	
	No negative effect on device (eg, drain phone battery)	264 (52.8)	
	Parts of the app can be used offline	256 (51.2)	
	People on the app have similar mental health experiences to mine	228 (45.6)	
**Most common activities participants would like to do using mental health apps**			
	Work through negative emotions and thoughts	329 (65.8)	
	Identify or recognize symptoms	290 (58)	
	Talk with other people to get/give support	244 (48.8)	
	Track symptoms	239 (47.8)	
	Distract myself from negative thoughts or emotions	228 (45.6)	
Stress score, mean (SD)			21.9 (5.3)^a^
Stigma score, mean (SD)			22.4 (6.2)^b^
Privacy score, mean (SD)			17.8 (7.6)^c^
Social influence score, mean (SD)			6.6 (3.4)^d^

^a^The score could range from 7 to 35.

^b^The score could range from 4 to 36.

^c^The score could range from 6 to 30.

^d^The score could range from 3 to 15.

### Barriers to Mental Health Resources, Important Aspects about Mental Health Apps, and Activities People Would Like to Do With Mental Health Apps

The community college studied provided some mental health resources, such as counseling and workshops on stress management. The most common barrier to accessing mental health resources was that participants preferred to deal with issues on their own (260/500, 52%). Other barriers were concerns about privacy (129/500, 25.8%), questioning how serious their needs were (126/500, 25.2%), and worries about what other people will think of them (116/500, 23.2%). The most important aspect, which was selected by 429 of the 500 participants (85.8%), was that the app was free. Other important aspects were that personal information would be kept private (397/500, 79.4%) and that people on the app had similar mental health experiences to theirs (228/500, 45.6%). In terms of what respondents would like to do with mental health resources, the most common answers were work through negative emotions and thoughts (329/500, 65.8%), identify or recognize symptoms (290/500, 58%), and talk with others to get or give support (244/500, 48.8%).

### Models to Understand Factors Associated with Mental Health App Use

Not all participants answered each survey question. After imputing missing values on continuous scale variables and excluding participants with missing data on dichotomous model variables (ie, perceived need and past use of services), the structural equation model included a subsample of 449 participants.

### Direct Effect Model

**Figure 1:**
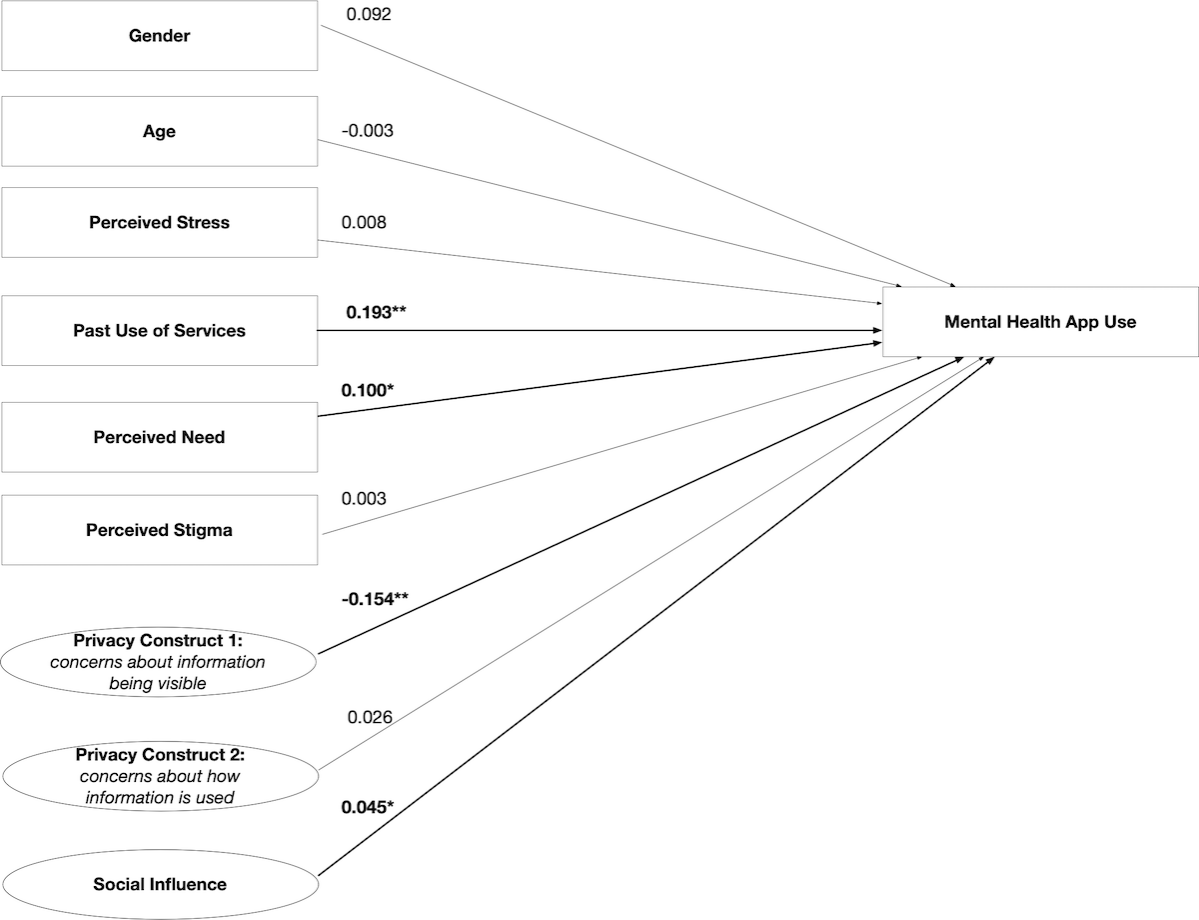
Direct effect model showing the path coefficients and levels of significance for relationships among variables. *Relationship is significant at *P*<.05; **relationship is significant at *P*<.01; n=449.

The standardized path coefficients of the direct effect model are shown in Figure 1. The model fit indices were χ^2^_87_=222.803, SRMR=0.089, and RMSEA=0.068. The adjusted *R*^2^ value of the model was 0.213.

### Full Mediation Model

**Figure 2:**
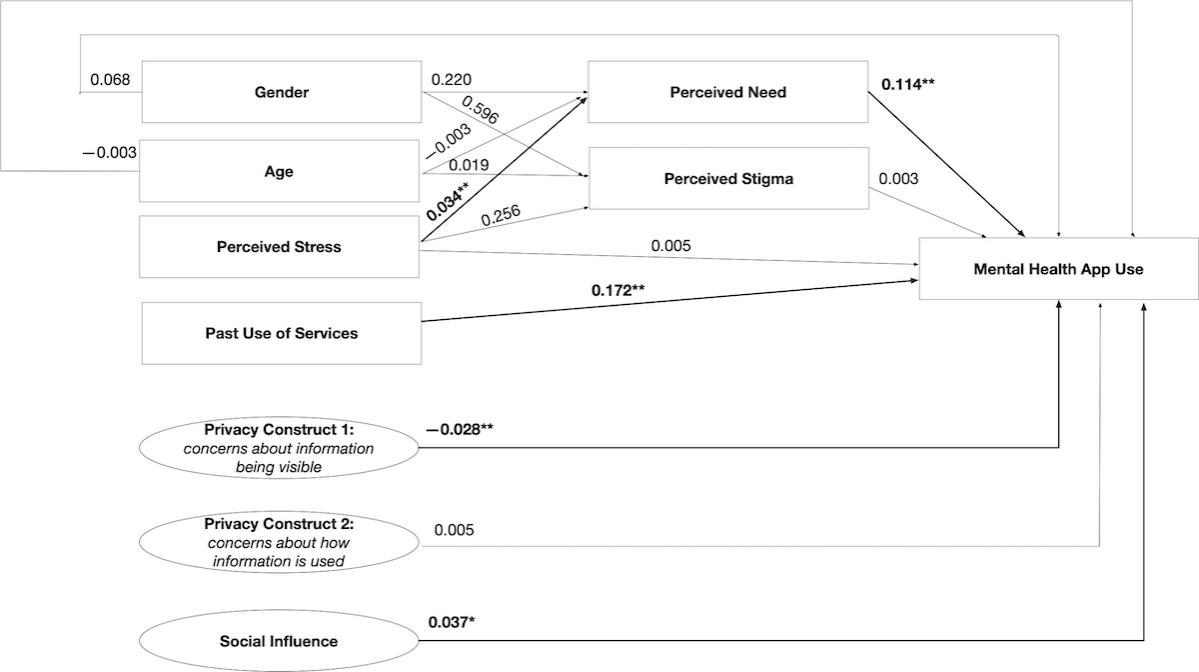
Full mediation model showing the path coefficients and levels of significance for relationships among variables. *Relationship is significant at *P*<.05; **relationship is significant at *P*<.01; n=449.

The standardized path coefficients of the full mediation model are shown in Figure 2. The model fit indices showed an acceptable model fit: χ^2^_89_=72.804, SRMR=0.051, and RMSEA=0.076. The adjusted *R*^2^ value of the model was 0.326. Table 5 compares the fit indices of the direct effect model and full mediation model, and the data indicate that the full mediation model had the best fit.

The model showed that past use of services, such as a counselor, was significantly associated with mental health app use. Perceived stigma had no effect on mental health app use. Some privacy concerns (ie, Privacy Construct 1) were associated with a lower likelihood of mental health app use. Social influence, measured by the importance participants placed on other people’s opinions on mental health app use, was associated with higher mental health app use. The effects of gender and age were nonsignificant.

**Table 5:** Fit indices of the direct effects model and full mediation model.

Statistic	Direct effects model	Full mediation model
Chi square (*df*)	222.803 (87)	72.804 (89)
SRMR^a^	0.089	0.051
RMSEA^b^	0.068	0.076
*R* ^2^	.213	.326

^a^SRMR: standardized root mean square residual.

^b^RMSEA: root mean square error of approximation.

The effect of perceived stress on the likelihood of mental health app use was mediated via perceived need. As Figure 2 illustrates, the regression coefficient between perceived stress and perceived need and the regression coefficient between perceived need and mental health app use were significant. The indirect effect was (0.034)*(0.114)=0.004. We used bootstrapping to test the significance of this indirect effect. Perceived need significantly mediated the effect of perceived stress on mental health app use (B=0.004, 95% CI 0.001 to 0.007; *P*=.01). There was no significant mediating effect of perceived stigma on the effect of perceived stress (B=0.001, 95% CI –0.002 to 0.011; *P*=.47).

## Discussion

### Principal Findings

The aim of this paper was to identify factors associated with mental health app use among community college students. The results revealed that participants’ use of mental health apps was associated with 5 factors: perceived stress, perceived need to seek help for mental health concerns, past use of professional mental health services, social influence of other people, and privacy concerns. Furthermore, the effect of stress was mediated by a perceived need to seek help. These findings support Hypotheses 1, 2, 4, and 6. Hypothesis 5 is partly supported, as only specific types of privacy concerns were negatively associated with app use. Hypothesis 3, which stated that the association of perceived stress and app use would be mediated by perceived stigma, was not supported by the results.

### Perceived Stress

Stress was listed as the most common mental health concern among participants (approximately 44% of participants experienced stress), and the results of the study indicate that the level of perceived stress predicted mental health app use. This means that the more stress people experienced, the more likely they were to have used apps. App use could therefore have been motivated by a student’s own perceived benefit of the apps in terms of their ability to reduce stress. Previous work has indicated a link between self-reported stress and interest in using a stress management app [21], as well as a link between perceived stress and a preference for technology over face-to-face mental health services [44]. Apolinário-Hagen et al [44] argue that people with stressful lives may find it easier to fit the use of a technology into their life than an in-person appointment, although further work is needed to test this assumption.

### Perceived Need to Seek Help

The results confirmed an association between a perceived need to seek help and app use, which extends previous work by showing that perceived need is not only a predictor of help-seeking through *nondigital* channels [3,23] but is also an important driver in using *digital* health interventions. The association indicates that students are more likely to have used mental health apps if they felt a need to seek help for concerns related to their mental health. In the current study, the effect of stress was mediated by a perceived need to seek help, which means that the effect of stress on mental health app use is even stronger if people not only experience stress, but also have a perceived need to seek help for issues related to mental health.

### Past Use of Professional Mental Health Services

Of the participants in our study, 23% had used professional mental health services in the past 12 months, which is broadly proportionate to the general community college population: a recent study analyzing survey data from 23 community colleges between 2016 and 2019 found that 30% of community college students had used therapy in the past year [45]. The community college studied also provided some on-campus mental health resources, such as counseling. Although the level of on-campus mental health support can vary per college, 73% of community colleges provide mental health counseling services on campus [46]. However, a previous study found that only a small percentage (5.4%) of community college students that seek help do so on campus [45]. This suggests that although offering resources is essential, it is imperative to understand potential barriers in accessing these resources. For example, campus counseling services often cannot keep up with the demand, which can cause students to feel undersupported [47,48].

In our study, past use of services was positively associated with mental health app use. For students that are already familiar with seeking help through nondigital channels, it may be less of a step to seek out help through a mental health app. However, it will likely matter how they experienced using mental health services, as people with a positive experience are generally more open to trying new mental health technologies [26-28], but people with a negative experience are less interested in mental health technology [49-51]. Future research should clarify the association between past use of professional mental health services and subsequent app use by assessing the quality of the previous experience with in-person services.

### Social Influence

The social influence construct was positively associated with mental health app use, which implies that if people close to the participants thought they should use mental health apps, they were more likely to use such apps. Technology use that is influenced by other people’s expectations of using it can create a sense of belonging [52]. Furthermore, people’s expectations could have been interpreted as them viewing the app as useful, as people are persuaded by messages from people close to them [35].

The effect of social influence in our model is in line with previous studies on mental health technologies, where engagement with a digital mental health program was facilitated by whether it was endorsed by friends and family [53] or the user’s current health care provider [54]. A survey among 102 university students and nonstudents found that one of the main reasons that participants used mental health technologies was that someone they knew recommended their use [55].

However, people can be deterred from using mental health technologies if they feel forced to use it by others, for example, if they feel pressure to use certain technologies from their health care provider [56]. It is therefore important that social influence happens organically through endorsement and recommendations rather than being prescribed top-down. Furthermore, a previous study that used a structural equation model to explain use of physical health apps found that social influence contributes to a decision to *initially* use an app but not to the intention to *continue* to use an app [57].

### Privacy

In our study, privacy played an important role in mental health app use, as supported by both the model and by privacy concerns being one of the most important aspects of and barriers to app use. Privacy of information is known to be an important aspect for students, with concerns about confidentiality of information [20] and data being leaked beyond the app [14]. A recent study reviewing 116 publicly available mobile apps for depression found that only a minority of these apps had a privacy policy and that most privacy policies were not transparent about how user data were collected, stored, and used [58]. A previous literature review of user engagement with mental health apps theorized that one reason for low user engagement with mental health apps is that many apps do not consider user privacy [59].

However, interestingly, only some aspects of privacy were significantly associated with app use in our model. Concerns related to personal information being potentially accessible by others was negatively associated with app use, meaning that people who had concerns about this aspect were less likely to use apps.

Privacy concerns about how personal information was subsequently used did not have an effect on app use. Privacy as a construct may be composed of different aspects that can impact mental health app use differently, which would be worthwhile to explore and refine further in future studies.

### Stigma

Consistent with a previous study among college students [3], the most common barrier to accessing mental health resources was that students preferred to deal with issues on their own, which may reflect stigma surrounding seeking help. Although over half of the participants had moderate to severe perceived stigma, this was not associated with mental health app use. Furthermore, our study did not find that the effect of stress on app use was mediated by stigma.

These findings suggest that whereas stigma can form a barrier to mental health help seeking in general [11,25], it may not bar people from using mental health apps in particular. In a previous study, participants who preferred digital over face-to-face mental health services even had higher levels of stigma [60]. When given the choice between digital and in-person services, digital resources may be a less stigmatizing option, although the current study did not provide support that a high level of stigma is necessarily associated with app use. Furthermore, our model included perceived stigma; however, stigma is multifaceted, and there are multiple other types of mental health stigma, such as internalized stigma and experienced stigma [61], that may affect use of mental health resources.

### Barriers to Using Mental Health Resources and Important Aspects of Mental Health Apps

Previous studies have highlighted potential differences in mental health concerns and needs among university and community college students. For example, whereas high workload and academic stress is a common issue raised among university students, financial or housing stress may be more common among community college students [9,10]. A previous study found that some community college students are uninsured and use web-based health information to avoid medical costs [10]. Our results show that the most important aspect of a mental health app was that it was free (85.8% of participants listed this as an important aspect). Furthermore, though the majority of participants (80.4%) had health insurance, only 24% were sure that their plan provided coverage for mental health services. It is thus important to address costs when considering mental health apps for community colleges.

Previous focus group studies have found differences in how technology is used, or would be used, by university and community college students for supporting their health. Whereas university students primarily use digital health resources in a preventive manner to recognize onset of symptoms [14], community college students tend to use digital health resources in a reactive manner when they are already experiencing symptoms [10]. Although these prior studies used small sample sizes and caution should be used in generalizing findings, they show there are different ways in which technologies may be used by different types of students. In our study, two-thirds of participants wanted to use mental health resources to work through negative emotions and thoughts, which further suggests a need for help with symptoms they are already experiencing.

### Implications

Previous work has shown that despite interest in mental health apps among students, use of these apps can be limited [20]. Our findings identify several factors associated with mental health app use among community college students. The results of this study can inform outreach and implementation of digital mental health resources on campuses. Below, we outline several implications that may be important to consider when implementing digital mental health resources for a community college student population.

#### Consider Factors That May Influence Engagement With Tools

Concerns about privacy were negatively associated with app use, and common barriers to accessing resources were related to costs and to students’ preference to deal with issues on their own. These barriers should be considered when offering digital resources to students. It is important to be transparent to students who will have access to their information. Furthermore, it is important to consider costs and, if possible, offer resources that are either low-cost or free to students. In addition, marketing mental health apps as self-guided tools that students can use to deal with issues on their own, rather than as a help-seeking service, may further facilitate use of mental health apps.

#### Mental Health Needs of Community College Students

It is important to think through specific preferences for accessing and integrating mental health support. Students who experienced stress, recognized a need to seek help, and had sought out help in the past were more likely to have used mental health apps. Counseling services can promote use of mental health apps among students already seeking help. A strength of technology is its availability [44] and the ability to access it anytime. Counselors can endorse particular apps to students so they can continue to have access to resources in addition to and after finishing counseling.

#### Endorsement and Use by Others May Help Mental Health App Adoption

For students who do not actively seek out help, it may not be sufficient to promote an app top-down through formal mental health services, although social influence of other people was positively associated with mental health app use. If other people have positive views about apps or personal experience with them, their endorsement may encourage other students to try an app. For outreach of apps, social platforms can be used to introduce apps, as seeing others sharing their experience and promoting an app may increase interest among students. In addition, the use of apps may be introduced and piloted in classrooms or other group settings, as the effect of social influence is strengthened if a technology is used in public rather than in private [62], and use by others may motivate students to use an app.

### Limitations and Future Work

This study has a number of limitations. First, the sample came from one community college, so caution must be taken in generalizing the results. We expect our findings to generalize to college student populations with similar demographics. Second, the results were collected during the COVID-19 pandemic and 1 month after the stay-at-home order took effect. Although we do not expect these circumstances to have influenced the relationships between stress and app use, it may have increased mental health concerns and increased interest in mental health apps. Given the cross-sectional nature of the survey, we do not have data available on changes in stress before and during the pandemic. Additionally, we focused on presenting and discussing a specific model to test the effects of 6 factors on mental health app use. There may be other mechanisms affecting app use, such as past experience with technology, which merit further exploration. To assess the factors of mental health app use and perceived need to seek help, single-item validated questions from prior work were used. Using short single item measures helps reduce participant burden of answering a long survey; however, it narrows the distribution of such variables. Lastly, the response rate was 12%; although this is a typical response rate for web-based surveys among student populations [2,5,20], there is potential for response bias, and there may be differences between nonresponders and responders. For example, although not all participants reported their race or ethnicity, the proportion of Hispanic/Latinx participants in our study sample was lower than that in the California community college population. Although classes at this community college are only taught in English, the English language questionnaire could have been a barrier to participation.

### Conclusions

This study focused on community college students and found 5 factors associated with mental health app use. Perceived stress, perceived need to seek help, and past use of professional services were positively associated with mental health app use. The more participants agreed that people in their social environment thought they should use apps, the more likely they were to use apps. Some privacy concerns were negatively associated with mental health app use. In addition, financial costs are an important aspect to consider for using mental health apps. The results can inform the selection and appropriate dissemination of mental health apps to meet college student needs.

## Appendix

Multimedia Appendix 1 Survey items.

## Abbreviations

CalMHSA: California Mental Health Service Authority
CFI: comparative fit index
RMSEA: root mean square error of approximation
SEM: structural equation modeling
SRMR: standardized root mean square residual
TAM: Technology Acceptance Model
TLI: Tucker-Lewis index
TRA: theory of reasoned action
UTAUT: Unified Theory of Acceptance and Use of Technology
