# Supplementary material for: Understanding Mental Health App Use Among Community College Students: Web-Based Survey Study
Source: J Med Internet Res. 2021 Sep 14;23(9):e27745. doi: 10.2196/27745 (PMC8479606; doi:10.2196/27745)
Supplement: Multimedia Appendix 1 [file jmir_v23i9e27745_app1.docx]

**Understanding How Mobile Technologies Can Address College Students’ Unmet Wellness and Mental Health Needs: Survey Items**

Q1 The purpose of this survey is to understand students’ mental health needs and the current strategies they use to manage mental health in order to provide appropriate resources (digital tools included) to students. Prior research shows that university and college students experience mental health concerns that often go undiagnosed and untreated, and that there is a need for increased access to mental health resources. However, research is lacking on community college students’ mental health needs, and the availability and use of technology in relation to these needs. We aim to address the following research questions:

(1) What are students’ current unmet wellness and mental health needs? 
(2) What are current tools and strategies used to manage health and wellness? 
(3) How can digital tools, such as apps, address unmet needs?
(4) What factors might influence adoption / engagement with mental health apps?

Q5 This part of the survey asks you about your background and demographic information. We use this information to understand who this data represents to make sure we best serve you and the community.

Q6 How old are you?

- Age: ________________________________________________
- I prefer not to answer.

Q7 What is your gender identity? (Select one answer.)

- Man
- Woman
- Transgender man
- Transgender woman
- Genderqueer / Gender non-conforming / Non-binary
- Questioning or unsure of gender identity
- Another gender identity ________________________________________________
- I prefer not to answer.

Q8 Please select the option that best describes your student enrollment status. (Select one answer.)

- Full-time student
- Part-time student
- Other (Please specify): ________________________________________________
- I prefer not to answer.

Q9 What best describes when you take the majority of classes at El Camino College? (Select one answer.)

- Daytime
- Evening
- Other (Please specify): ________________________________________________
- I prefer not to answer.

Q10 What is your race? (Select one answer.)

- American Indian or Alaska Native
- Asian
- Black or African American
- Native Hawaiian or other Pacific Islander
- White
- Other: ________________________________________________
- More than one race
- I prefer not to answer.

Q11 What is your ethnicity? (Select one answer. If you are multi-ethnic, please check "more than one ethnicity.")

- Caribbean
- Central American
- Mexican/Mexican-American/Chicano
- Puerto Rican
- South American
- Other ________________________________________________
- African
- Asian Indian/South Asian
- Cambodian
- Chinese
- Eastern European
- European
- Filipino
- Japanese
- Korean
- Middle Eastern
- Vietnamese
- Other ________________________________________________
- More than one ethnicity
- I prefer not to answer.

Q12 What is your sexual orientation? (Select one answer.)

- Gay or Lesbian
- Heterosexual or Straight
- Bisexual
- Questioning or unsure of sexual orientation
- Queer
- Another sexual orientation: ________________________________________________
- I prefer not to answer.

Q13 What language do you most often speak at home? (Select one answer.)

- Arabic
- Armenian
- Cambodian
- Cantonese
- English
- Farsi
- Hmong
- Korean
- Mandarin
- Other Chinese
- Russian
- Spanish
- Tagalog
- Vietnamese
- American Sign Language
- Other: ________________________________________________
- I prefer not to answer.

End of Block: Demographics pt. 1

Start of Block: General technology and smartphone use

Q14 This section is about your technology and smartphone use.

Q15 Which of the following do you use? (Select all that apply.)

- Desktop or laptop computer
- Smartphone
- Tablet (e.g., iPad)
- Mobile phone or cell phone but not a smartphone
- I don't use any of these.
- I prefer not to answer.

Display This Question:

If Which of the following do you use? (Select all that apply.) = Desktop or laptop computer

Or Which of the following do you use? (Select all that apply.) = Smartphone

Or Which of the following do you use? (Select all that apply.) = Tablet (e.g., iPad)

Q16 On a typical day, how often do you use a computer or mobile device for social media? (Select one answer.)
 
*Social media may include Facebook, Instagram, Twitter, Snapchat, Youtube, etc.*

- Almost constantly
- Many times a day
- A few times a day
- Less than a few times a day
- I prefer not to answer.

Q17 People may use the internet for streaming video/music, playing games, checking social media, using apps, browsing the web, etc. on a computer or on a phone or mobile device.
 
On a typical day, how often do you use the internet? (Select one answer.)

- Almost constantly
- Many times a day
- A few times a day
- Less than a few times a day
- I prefer not to answer.

Q18 Where do you most often access internet? (Select one answer.)

- At home
- At work
- On school campus
- Other ________________________________________________
- I prefer not to answer.

Q19 Do you have consistent access to WiFi? (Select one answer.)

- Yes
- No
- I’m not sure.
- I prefer not to answer.

Display This Question:

If Which of the following do you use? (Select all that apply.) = Smartphone

Q20 If you have a smartphone, what do you use it for? (Select all that apply.)

- Communication
- Entertainment
- Social media
- Email
- School/homework
- News/current events
- Travel information/navigation
- Nutrition/diet
- Fitness/Exercise
- Mental health
- Other ________________________________________________
- I prefer not to answer.

Q21 Do you have a mobile data plan? (Select one answer.)

- Yes
- No
- I'm not sure.
- I prefer not to answer.

Display This Question:

If Which of the following do you use? (Select all that apply.) = Smartphone

Q22 If you have a smartphone, are you concerned about having enough space to download apps? (Select one answer.)

- Yes
- No
- I'm not sure.
- I prefer not to answer.

Display This Question:

If Do you have a mobile data plan? (Select one answer.) = Yes

Q23 Do you have concerns about your mobile data plan when using your phone (for example, concerns that you will run out of data, an app will use up a lot of your data, etc.)? (Select one answer.)

- Yes
- No
- I'm not sure.
- I prefer not to answer.

End of Block: General technology and smartphone use

Start of Block: Mental health apps

Q24 This section is about technology specifically for mental health.

Q25 Have you ever used a mental health app? (Select one answer.)   *By mental health app, we mean an application on your mobile phone or tablet device that helps you manage your mental, emotional, or psychological health or get access to resources to support your mental, emotional, or psychological health.*

- Yes, I currently use a mental health app.
- Yes, I have used a mental health app but no longer use one.
- No, I have never used a mental health app, but I'm interested in using one.
- No, I have never used a mental health app, and I don't want to use one.
- I prefer not to answer.

Display This Question:

If Have you ever used a mental health app? (Select one answer.)   By mental health app, we mean an a... = Yes, I currently use a mental health app.

Or Have you ever used a mental health app? (Select one answer.)   By mental health app, we mean an a... = Yes, I have used a mental health app but no longer use one.

Q26 The next set of statements are going to ask you about your views on using mental health apps. For each statement, please rate the extent to which you agree or disagree, using a scale from **Strongly disagree (1) to Strongly agree (5).**

|  | Strongly disagree- 1 | Somewhat disagree- 2 | Neither agree nor disagree- 3 | Somewhat agree- 4 | Strongly agree- 5 | I prefer not to answer. |
| --- | --- | --- | --- | --- | --- | --- |
| I find mental health apps useful in my daily life. |  |  |  |  |  |  |
| Using mental health apps increases my chances of achieving things that are important to me. |  |  |  |  |  |  |
| Using mental health apps helps me accomplish things more quickly. |  |  |  |  |  |  |
| Using mental health apps increases my productivity. |  |  |  |  |  |  |

Q27 The next set of statements are going to ask you about your views on mental health apps. For each statement, please rate the extent to which you agree or disagree, using a scale from **Strongly disagree (1) to Strongly agree (5)**.

|  | Strongly disagree- 1 | Somewhat disagree- 2 | Neither agree nor disagree- 3 | Somewhat agree- 4 | Strongly agree- 5 | I prefer not to answer. |
| --- | --- | --- | --- | --- | --- | --- |
| People who are important to me think that I should use mental health apps. |  |  |  |  |  |  |
| People who influence my behavior think that I should use mental health apps. |  |  |  |  |  |  |
| People whose opinions I value prefer that I use mental health apps. |  |  |  |  |  |  |
| I have the knowledge necessary to use mental health apps. |  |  |  |  |  |  |
| I have the resources necessary to use mental health apps. |  |  |  |  |  |  |
| Mental health apps are compatible with other technologies I use. |  |  |  |  |  |  |
| I can get help from others when I have difficulties using mental health apps. |  |  |  |  |  |  |

Display This Question:

If Have you ever used a mental health app? (Select one answer.)   By mental health app, we mean an a... = Yes, I currently use a mental health app.

Or Have you ever used a mental health app? (Select one answer.)   By mental health app, we mean an a... = Yes, I have used a mental health app but no longer use one.

Q28 The next set of statements are going to ask you about your views on how your personal information might be used by a mental health app. For each statement, please rate the extent to which you agree or disagree on a scale from **Strongly disagree (1) to Strongly agree (5)**.

|  | Strongly disagree-1 | Somewhat disagree-2 | Neither agree nor disagree-3 | Somewhat agree-4 | Strongly agree-5 | I prefer not to answer. |
| --- | --- | --- | --- | --- | --- | --- |
| I feel that as a result of my using mental health apps, others know about me more than I am comfortable with. |  |  |  |  |  |  |
| I believe that as a result of my using mental health apps, information about me that I consider private is now more readily available to others than I would want. |  |  |  |  |  |  |
| I feel that as a result of my using mental health apps, information about me is out there that, if used, will invade my privacy. |  |  |  |  |  |  |
| I am concerned that mental health apps may use my personal information for other purposes without notifying me or getting my authorization. |  |  |  |  |  |  |
| When I give personal information to use mental health apps, I am concerned that it may use my information for other purposes. |  |  |  |  |  |  |
| I am concerned that mental health apps may share my personal information with other groups without getting my authorization. |  |  |  |  |  |  |

Display This Question:

If Have you ever used a mental health app? (Select one answer.)   By mental health app, we mean an a... = No, I have never used a mental health app, but I'm interested in using one.

Or Have you ever used a mental health app? (Select one answer.)   By mental health app, we mean an a... = No, I have never used a mental health app, and I don't want to use one.

Q29 The next set of statements are going to ask you about your views on how your personal information might be used by a mental health app. For each statement, please rate the extent to which you agree or disagree on a scale from **Strongly disagree (1) to Strongly agree (5)**.

|  | Strongly disagree-1 | Somewhat disagree-2 | Neither agree nor disagree-3 | Somewhat agree-4 | Strongly agree-5 | I prefer not to answer. |
| --- | --- | --- | --- | --- | --- | --- |
| I feel that if I were to use mental health apps, others know about me more than I am comfortable with. |  |  |  |  |  |  |
| I believe that if I were to use mental health apps, information about me that I consider private is now more readily available to others than I would want. |  |  |  |  |  |  |
| I feel that if I were to use mental health apps, information about me is out there that, if used, will invade my privacy. |  |  |  |  |  |  |
| I am concerned that mental health apps may use my personal information for other purposes without notifying me or getting my authorization. |  |  |  |  |  |  |
| If I were to give personal information to use mental health apps, I am concerned that it may use my information for other purposes. |  |  |  |  |  |  |
| I am concerned if I were to use mental health apps that they may share my personal information with other groups without getting my authorization. |  |  |  |  |  |  |

Q30 When thinking about using mental health apps, what other aspects are important to you? (Select all that apply.)

- Availability in languages other than English
- The app is free
- The app is sensitive to my culture
- People I interact with on the app share the same cultural background as I do
- People I interact with on the app share similar mental health experience as I do
- My personal information will be kept private
- The app will not have a negative effect on my device (examples: using the app will not drain my phone battery, using the app will not take up too much memory)
- Parts of the app can be used offline
- The app can be easily used by people with visual impairments
- The app can be easily used by people who are deaf or hard of hearing
- Other (Please specify) ________________________________________________
- I prefer not to answer.

End of Block: Mental health apps

Start of Block: Healthcare utilization and resources

Q31 This section is about healthcare utilization and resources.

Q32 Do you currently have health insurance? (Select one answer.)

- Yes
- No
- I prefer not to answer.

Display This Question:

If Do you currently have health insurance? (Select one answer.) = Yes

Q33 Do you know if your health insurance plan would provide any coverage for a visit to a mental health professional (psychiatrist, psychologist, clinical social worker, etc.)? (Select one answer.)

- I have no idea.
- I think it would not, but I am not sure.
- No, it definitely would not.
- Yes, it definitely would.
- I think it would, but I am not too sure.
- I prefer not to answer.

Q34 Was there ever a time during the past 12 months when you felt that you might need to see a professional because of problems with your mental health, emotions, or nerves or your use of alcohol or drugs? (Select one answer.)

- Yes
- No
- I don't know
- I prefer not to answer

Display This Question:

If Was there ever a time during the past 12 months when you felt that you might need to see a profes... = Yes

Or Was there ever a time during the past 12 months when you felt that you might need to see a profes... = I don't know

Q35 What mental health concerns, if any, have you experienced in the past 12 months? (Select all that apply.) If these do not capture your mental health concerns, then please feel free to write your thoughts in your own words. *If you prefer not to answer, then please select "I prefer not to answer."*

- Depression
- Anxiety
- Stress
- Difficulty sleeping
- Loneliness
- Substance and/or alcohol abuse
- Eating disorder
- Obsessions and compulsions that interfere with daily activities
- Mental health concerns after giving birth
- Dealing with a life event (e.g., moving, death, illness)
- Interpersonal relationships (e.g., broke up with significant other, had a fight with parent)
- Other (please specify): ________________________________________________
- I prefer not to answer.
- I haven't experienced any mental health concerns in the past 12 months.

Q36 In the past 12 months, have you seen your **primary care physician or general practitioner** for problems with your mental health, emotions, or nerves or your use of alcohol or drugs? (Select one answer.)

- Yes
- No
- I don't know
- I prefer not to answer.

Q37 In the past 12 months, have you seen **any other professional, such as a counselor, psychiatrist, or social worker**, for problems with your mental health, emotions, or nerves or your use of alcohol or drugs?

- Yes
- No
- I don't know
- I prefer not to answer

Display This Question:

If In the past 12 months, have you seen your primary care physician or general practitioner for prob... = Yes

Or In the past 12 months, have you seen any other professional, such as a counselor, psychiatrist, o... = Yes

Q38 Did you seek help for your mental or emotional health OR for an alcohol or drug problem?

- Yes, for mental or emotional health
- Yes, for an alcohol or drug problem
- Yes, for both mental or emotional health AND an alcohol or drug problem
- I don’t know.
- I prefer not to answer.

Display This Question:

If In the past 12 months, have you seen your primary care physician or general practitioner for prob... = Yes

Or In the past 12 months, have you seen any other professional, such as a counselor, psychiatrist, o... = Yes

Q39 Are you still receiving treatment for these problems from one or more of these healthcare providers?

- Yes
- No
- I don't know.
- I prefer not to answer

Q40 In the past 12 months, have you tried to get help from an online tool, including mobile apps or texting services for problems with your mental health, emotions, nerves, or your use of alcohol or drugs? (Select one answer.)

- Yes
- No
- I prefer not to answer.

Display This Question:

If In the past 12 months, have you tried to get help from an online tool, including mobile apps or t... = Yes

Q41 How useful was this online tool?  (Select one answer.)

- Very
- Somewhat
- Not at all
- I prefer not to answer.

Display This Question:

If In the past 12 months, have you tried to get help from an online tool, including mobile apps or t... = No

Q42 What was the main reason you didn't try to get help from an online tool, including mobile apps or texting services? (Select one answer.)

- Got better / no longer needed
- Wanted to handle problem myself
- Don't own a smartphone or computer
- Didn't know about these apps
- Don't trust mobile apps
- Concerns about privacy and security of data
- Don't think it would be helpful or work
- Cost
- Don't have time
- Received traditional / face-to-face services
- Don't think I needed it
- Don't have enough space to download new apps
- Other (Please specify): ________________________________________________
- I prefer not to answer.

Q43 In the past 12 months, have you connected online with people that have mental health or alcohol/drug concerns similar to yours through methods such as social media, blogs, and online forums? (Select one answer.)

- Yes
- No
- I prefer not to answer.

Q44 In the past 12 months, have you used online tools to find, be referred to, contact, or connect with a mental health professional? (Select one answer.)
*For example, by texting, online messaging, video chat, or a mental health or health-related mobile app*

- Yes
- No
- I prefer not to answer.

Q45 If you needed to seek resources for your mental health while attending school, would you know where to go? (Select one answer.)

- Yes
- No
- I prefer not to answer.

Q46 What resources have you used at El Camino College? (Select all that apply.)

- Psychological counseling
- Mental Health Wellness Workshops (examples: anxiety workshop, finding balance, substance abuse, perfectly imperfect)
- De-Stress For Survival Group
- Classroom Workshops (examples: stress management, time management, healthy relationships, avoiding burnout/self-care)
- Other (Please specify): ________________________________________________
- I have not used any resources at El Camino College.
- I prefer not to answer.

Q47 Who do you usually talk to when you feel sad, anxious, worried, or stressed? (Select all that apply.)

- A friend
- A family member
- Professor or instructor
- Teaching Assistant
- Academic counselor
- Staff at Student Health Center
- Off-campus medical center
- I don't talk to anyone.
- Other (please specify): ________________________________________________
- I prefer not to answer.

Q48 Which of the following resources and strategies do you **currently use** to manage your mental health? (Select all that apply).

- Informal support, such as talking with or spending time with family or friends
- Professional services (examples: counseling with psychologist, clinical social worker, psychiatrist)
- Social media (examples: Facebook, Twitter, Reddit)
- Online forums or communities (examples: Mental Health Forum, BeyondBlue)
- Websites (examples: Moodgym, Psychology Today)
- Mobile apps (examples: 7 Cups, Headspace, Moodpath)
- ACCESS Line, which serves as the primary entry point for mental health services with the Los Angeles County Department of Mental Health
- Other phone-based or text-based crisis lines (e.g., Crisis Text Line, Suicide Prevention Lifeline)
- Exercise programs or physical activities
- Writing
- Painting, drawing, coloring, photography, etc.
- Making crafts, sewing, etc.
- Listening to music
- Playing an instrument, singing, or making music
- Reading
- Cooking or baking
- Playing games
- Other (Please Specify): ________________________________________________
- I don't currently use any resources or strategies to manage my mental health.
- I prefer not to answer.

Q91 Which of the following resources and strategies **would you like to use** to manage your mental health? (Select all that apply).

- Informal support, such as talking with or spending time with family or friends
- Professional services (examples: counseling with psychologist, clinical social worker, psychiatrist)
- Social media (examples: Facebook, Twitter, Reddit)
- Online forums or communities (examples: Mental Health Forum, BeyondBlue)
- Websites (examples: Moodgym, Psychology Today)
- Mobile apps (examples: 7 Cups, Headspace, Moodpath)
- ACCESS Line, which serves as the primary entry point for mental health services with the Los Angeles County Department of Mental Health
- Other phone-based or text-based crisis lines (e.g., Crisis Text Line, Suicide Prevention Lifeline)
- Exercise programs or physical activities
- Writing
- Painting, drawing, coloring, photography, etc.
- Making crafts, sewing, etc.
- Listening to music
- Playing an instrument, singing, or making music
- Reading
- Cooking or baking
- Playing games
- Other (Please Specify): ________________________________________________
- I am not interested in using any resources or strategies to manage my mental health.
- I prefer not to answer.

Display This Question:

If Which of the following resources and strategies do you currently use to manage your mental health... != I prefer not to answer.

And Which of the following resources and strategies do you currently use to manage your mental health... != I don't currently use any resources or strategies to manage my mental health.

Carry Forward Selected Choices from "Which of the following resources and strategies do you currently use to manage your mental health? (Select all that apply)."

| 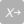 |
| --- |

Q49 For each resource you currently use, please rate how useful that resource is in supporting your mental health, on a scale from **Not at all useful (1) to Extremely useful (5).**

|  | Not at all useful-1 | Slightly useful-2 | Moderately useful-3 | Very useful-4 | Extremely useful-5 | I prefer not to answer. |
| --- | --- | --- | --- | --- | --- | --- |
| Informal support, such as talking with or spending time with family or friends |  |  |  |  |  |  |
| Professional services (examples: counseling with psychologist, clinical social worker, psychiatrist) |  |  |  |  |  |  |
| Social media (examples: Facebook, Twitter, Reddit) |  |  |  |  |  |  |
| Online forums or communities (examples: Mental Health Forum, BeyondBlue) |  |  |  |  |  |  |
| Websites (examples: Moodgym, Psychology Today) |  |  |  |  |  |  |
| Mobile apps (examples: 7 Cups, Headspace, Moodpath) |  |  |  |  |  |  |
| ACCESS Line, which serves as the primary entry point for mental health services with the Los Angeles County Department of Mental Health |  |  |  |  |  |  |
| Other phone-based or text-based crisis lines (e.g., Crisis Text Line, Suicide Prevention Lifeline) |  |  |  |  |  |  |
| Exercise programs or physical activities |  |  |  |  |  |  |
| Writing |  |  |  |  |  |  |
| Painting, drawing, coloring, photography, etc. |  |  |  |  |  |  |
| Making crafts, sewing, etc. |  |  |  |  |  |  |
| Listening to music |  |  |  |  |  |  |
| Playing an instrument, singing, or making music |  |  |  |  |  |  |
| Reading |  |  |  |  |  |  |
| Cooking or baking |  |  |  |  |  |  |
| Playing games |  |  |  |  |  |  |
| Other (Please Specify): |  |  |  |  |  |  |
| I don't currently use any resources or strategies to manage my mental health. |  |  |  |  |  |  |
| I prefer not to answer. |  |  |  |  |  |  |

Display This Question:

If Which of the following resources and strategies would you like to use to manage your mental healt... != I prefer not to answer.

And Which of the following resources and strategies would you like to use to manage your mental healt... != I am not interested in using any resources or strategies to manage my mental health.

Carry Forward Selected Choices from "Which of the following resources and strategies would you like to use to manage your mental health? (Select all that apply)."

| 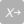 |
| --- |

Q50 For each resource you'd like to use but don't currently use, please rate how likely you are to use that resource on a scale from **Extremely unlikely (1) to Extremely likely (5).**

|  | Extremely unlikely-1 | Somewhat unlikely-2 | Neither likely nor unlikely-3 | Somewhat likely-4 | Extremely likely-5 |
| --- | --- | --- | --- | --- | --- |
| Informal support, such as talking with or spending time with family or friends |  |  |  |  |  |
| Professional services (examples: counseling with psychologist, clinical social worker, psychiatrist) |  |  |  |  |  |
| Social media (examples: Facebook, Twitter, Reddit) |  |  |  |  |  |
| Online forums or communities (examples: Mental Health Forum, BeyondBlue) |  |  |  |  |  |
| Websites (examples: Moodgym, Psychology Today) |  |  |  |  |  |
| Mobile apps (examples: 7 Cups, Headspace, Moodpath) |  |  |  |  |  |
| ACCESS Line, which serves as the primary entry point for mental health services with the Los Angeles County Department of Mental Health |  |  |  |  |  |
| Other phone-based or text-based crisis lines (e.g., Crisis Text Line, Suicide Prevention Lifeline) |  |  |  |  |  |
| Exercise programs or physical activities |  |  |  |  |  |
| Writing |  |  |  |  |  |
| Painting, drawing, coloring, photography, etc. |  |  |  |  |  |
| Making crafts, sewing, etc. |  |  |  |  |  |
| Listening to music |  |  |  |  |  |
| Playing an instrument, singing, or making music |  |  |  |  |  |
| Reading |  |  |  |  |  |
| Cooking or baking |  |  |  |  |  |
| Playing games |  |  |  |  |  |
| Other (Please Specify): |  |  |  |  |  |
| I am not interested in using any resources or strategies to manage my mental health. |  |  |  |  |  |
| I prefer not to answer. |  |  |  |  |  |

Q58 When thinking about using mental health resources, what things would you like to be able to do? (Select all that apply.)

- Identify or recognize symptoms
- Track symptoms
- Work through negative emotions and thoughts
- Talk with other people to get / give support
- Connect with a mental health professional
- Read mental health experiences of other people
- Stay organized and keep on track of tasks and responsibilities
- Express myself or have an outlet through art, photos, or writing
- Distract myself from negative thoughts or emotions
- Get information about mental health symptoms and conditions
- Get information about how to access local mental health resources
- Access educational materials on how to cope with stress
- Get information about how to handle grief or loss
- Get information about how to deal with trauma
- Get information about how to handle relationship issues
- Relieve stress, worry, or anxiety related specifically to coronavirus (also known as COVID-19)
- Other (Please specify): ________________________________________________
- I prefer not to answer.

Q59
What barriers, if any, do you face to accessing mental health-related resources? (Select all that apply.)

- I prefer to deal with issues on my own
- There are financial reasons (too expensive, no insurance)
- I don’t have time
- The waiting time to access resources is too long
- I am concerned about privacy
- I worry that my actions will be documented
- I worry about what others will think of me
- I worry that someone will notify my parents
- People providing services aren’t sensitive enough to cultural differences
- People providing services aren’t sensitive enough to sexual identity differences
- I have a hard time communicating in English
- I question the quality of my options
- I question whether the resources are helpful
- I have had a bad experience with these resources in the past
- The problem will get better by itself
- I question how serious my needs are
- I don’t think anyone can understand my problems
- Stress is normal at community college
- I get a lot of support from other sources
- There have been no barriers or challenges that I can think of
- I have not had any need for resources
- Other (Please specify): ________________________________________________
- I prefer not to answer.

Q60 When do you need support the most? (Select all that apply.)

- During the day
- At night
- At home
- At school
- At work
- Before or after a difficult exam
- Before or during a social situation
- When dealing with a significant life event (e.g., after a breakup, death or illness in my family)
- Other (Please specify): ________________________________________________
- I prefer not to answer.

End of Block: Healthcare utilization and resources

Start of Block: Stress, well-being, and mental health experiences

Q61
This section is about stress, well-being, and mental health.

Q62 The next set of statements are going to ask you about how often you experience stress in the following situations. For each statement, please rate how frequently you are distressed or anxious, on a scale from **Never (1) to Very often (5)**.

|  | Never- 1 | Rarely- 2 | Sometimes- 3 | Often- 4 | Very often- 5 | I prefer not to answer. |
| --- | --- | --- | --- | --- | --- | --- |
| Personal relationships |  |  |  |  |  |  |
| Family matters |  |  |  |  |  |  |
| Financial matters |  |  |  |  |  |  |
| Academic matters |  |  |  |  |  |  |
| Housing matters |  |  |  |  |  |  |
| Being away from home |  |  |  |  |  |  |
| Events not going as planned |  |  |  |  |  |  |

Q63 The next questions are about the one month in the past 12 months when you were at your worst emotionally.    During that same month, did your emotions interfere **a lot (1), some (2), or not at all (3)** with…

|  | A lot-1 | Some-2 | Not at all-3 | Not applicable | I prefer not to answer. |
| --- | --- | --- | --- | --- | --- |
| …your performance at school? |  |  |  |  |  |
| …your performance at work? |  |  |  |  |  |
| …your household chores? |  |  |  |  |  |
| …your social life? |  |  |  |  |  |
| …your relationship with friends and family? |  |  |  |  |  |

Q64 The next set of statements are going to ask you about how you feel about different aspects of your life. For each statement, please rate how often you feel that way, on a scale from **Hardly ever (1) to Often (3)**.

|  | Hardly ever-1 | Some of the time-2 | Often-3 | I prefer not to answer. |
| --- | --- | --- | --- | --- |
| How often do you feel that you lack companionship? |  |  |  |  |
| How often do you feel left out? |  |  |  |  |
| How often do you feel isolated from others? |  |  |  |  |

Q65 The next set of statements are going to ask you about how you have been feeling during the past 30 days. For each statement, please rate how often you have been feeling that way over the past 30 days, on a scale from **None of the time (1) to All of the time (5)**.  
 
During the last 30 days, about how often did you feel...

|  | None of the time-1 | A little of the time-2 | Some of the time-3 | Most of the time-4 | All of the time-5 | I prefer not to answer. |
| --- | --- | --- | --- | --- | --- | --- |
| ...tired out for no good reason? |  |  |  |  |  |  |
| ...nervous? |  |  |  |  |  |  |
| ...so nervous that nothing could calm you down? |  |  |  |  |  |  |
| ...hopeless? |  |  |  |  |  |  |
| ...restless or fidgety? |  |  |  |  |  |  |
| ...so restless you could not sit still? |  |  |  |  |  |  |
| ...depressed? |  |  |  |  |  |  |
| ...that everything was an effort? |  |  |  |  |  |  |
| ...so sad that nothing could cheer you up? |  |  |  |  |  |  |
| ...worthless? |  |  |  |  |  |  |

End of Block: Stress, well-being, and mental health experiences

Start of Block: Mental Health Perceptions

Q66 Many people have experienced (or are currently experiencing) a mental illness that impacted their ability to function in their family, relationships, or workplace. Many different terms are used to refer to those experiences - some terms include "mental illness," "mental health problem," "emotional distress," "psychological disorder," "mental challenge," etc.
 
Have you ever experienced a mental illness? (Select one answer.)

- Yes, currently or previously
- No, never
- I prefer not to answer.

Display This Question:

If Many people have experienced (or are currently experiencing) a mental illness that impacted their... = Yes, currently or previously

Q67 This part of the survey looks at mental health stigma. Stigma can cause people to feel badly for something that is out of their control. Keep in mind, these statements **don’t**represent our views of mental health or mental illness. 

 Throughout this section, the term “mental illness” will be used. However, there are many different terms that could be used, such as mental health, mental disorder, mental health condition, psychological health, emotional well-being, to name a few. 

 Please rate the extent to which you agree on a scale from**Strongly disagree (1) to Strongly agree (4)** for the following statements.

|  | Strongly disagree- 1 | Disagree- 2 | Agree- 3 | Strongly agree- 4 | I prefer not to answer. |
| --- | --- | --- | --- | --- | --- |
| Stereotypes about people with a mental illness apply to me. |  |  |  |  |  |
| In general, I am able to live life the way I want to. |  |  |  |  |  |
| Negative stereotypes about the mentally ill keep me isolated from the ‘normal’ world. |  |  |  |  |  |
| I feel out of place in the world because I have a mental illness. |  |  |  |  |  |
| Being around people who don’t have a mental illness makes me feel out of place or inadequate. |  |  |  |  |  |
| People without a mental illness could not possibly understand me. |  |  |  |  |  |
| Nobody would be interested in getting close to me because I have a mental illness. |  |  |  |  |  |
| I can’t contribute anything to society because I have a mental illness. |  |  |  |  |  |
| I can have a good, fulfilling life, despite my mental illness. |  |  |  |  |  |
| In my culture, it's acceptable to get treatment for mental illness. |  |  |  |  |  |

Q68 The following questions are going to ask you about your personal thoughts and opinions about **those with a mental illness.** Throughout this section, the term "mental illness" will be used. However, there are other terms that could be used, such as mental disorder, psychological disorder, or mental health condition. Please rate the extent to which you agree, on a scale from **Strongly Disagree (1) to Strongly Agree (4)** for the following statements.

|  | Strongly disagree-1 | Disagree-2 | Agree-3 | Strongly agree-4 | I prefer not to answer. |
| --- | --- | --- | --- | --- | --- |
| Most people believe that people with a mental illness could snap out of it if they wanted. |  |  |  |  |  |
| Most people believe that having a mental illness is a sign of personal weakness. |  |  |  |  |  |
| Most people believe that a mental illness is not a real medical illness. |  |  |  |  |  |
| Most people believe that people with a mental illness are dangerous. |  |  |  |  |  |
| Most people believe that it is best to avoid people with a mental illness so you don't become mentally ill yourself. |  |  |  |  |  |
| Most people believe that people with mental illness are unpredictable. |  |  |  |  |  |
| If they had a mental illness, most people would not tell anyone. |  |  |  |  |  |
| Most people would not employ someone they knew had a mental illness. |  |  |  |  |  |
| Most people would not vote for a politician they knew had a mental illness. |  |  |  |  |  |

Q69 What else would you like us to know? This includes but is not limited to sharing more information about your mental health or wellness, coping strategies and resources, or use of and interest in technology? *If you prefer not to answer, then you can leave this blank.*

________________________________________________________________

End of Block: Mental Health Perceptions

Start of Block: Demographics pt. 2

Q70 The part of the survey asks you about your background and demographic information. We use this information to understand who this data represents to make sure we best serve you and the community.

Q71 Please select the option that best describes your employment status. (Select one answer.)

- Employed full time
- Employed part time
- Unemployed looking for work
- Unemployed not looking for work
- Retired
- Disabled
- I prefer not to answer.
- Other (Please specify): ________________________________________________

Q72 Please select the option that best describes your current marital status. (Select one answer.)

- Single
- In a committed relationship or partnership but unmarried
- Married
- Widowed
- Divorced
- Separated
- Other (Please specify): ________________________________________________
- I prefer not to answer.

Q73 Do you have children or dependents? (Select one answer.)

- Yes
- No
- I prefer not to answer.

Q74 What is your current living situation? (Select one answer.)

- I live alone.
- I live with a spouse or partner.
- I live with roommate(s).
- I live with my children.
- I live with family.
- Other ________________________________________________
- I prefer not to answer.

Q75 Are you currently experiencing homelessness? (Select one answer.)

- Yes
- No
- I prefer not to answer.

Q76 What is your current annual household income? (Select one answer.)

- Under $10,000
- $10,000-$19,999
- $20,000-$29,999
- $30,000-$39,999
- $40,000-$49,999
- $50,000-$59,999
- $60,000-$69,999
- $70,000-$79,999
- $80,000-$89,999
- $90,000-$99,999
- $100,000-$149,999
- $150,000 or above
- I prefer not to answer.

Q77 Are you a veteran? (Select one answer.)

- Yes
- No
- I prefer not to answer.

Q78 Do you have a disability? (Select one answer.)
   For this questionnaire, disability is defined as a mental or physical impairment lasting more than 6 months and limiting major life activity but is not the result of a severe mental illness.

- Yes
- No
- I prefer not to answer.

Display This Question:

If Do you have a disability? (Select one answer.)   For this questionnaire, disability is defined as... = Yes

Q79 What type of disability do you have? (Select all that apply.) For this questionnaire, disability is defined as a mental or physical impairment lasting more than 6 months and limiting major life activity but is not the result of a severe mental illness.

- A mental disability
- A physical/mobility disability
- A chronic health condition (including chronic pain)
- Difficulty seeing
- Difficulty hearing
- Other disability (Please specify): ________________________________________________
- I prefer not to answer.

Q80 This last part of the survey will ask questions related to how coronavirus/COVID-19 has impacted you.

Q81 Have you or anyone you know been diagnosed with the coronavirus/COVID-19? (Select one answer.).

- Yes
- No
- I prefer not to answer.

Display This Question:

If Have you or anyone you know been diagnosed with the coronavirus/COVID-19? (Select one answer.). = Yes

Q82 Who has been diagnosed? (Select all that apply.)

- Self
- Partner/spouse
- Child
- Parent
- Grandparent
- Friend
- Colleague
- Acquaintance
- Other (Please specify): ________________________________________________
- I prefer not to answer.

Q83 Have you had a change in your employment because of the coronavirus/COVID-19? (Select one answer.)

- Lost my job
- Had my hours reduced
- Same job and hours, but working less due to children at home/other caregiving responsibilities
- Working from home/remotely - my choice
- Working from home/remotely - required by employment/government mandate
- No change in my employment
- I prefer not to answer.

Q84 Please describe the economic impact on your family (Select all that apply.)

- Another member of the family has lost their job
- Another member of the family has had their hours reduced
- Another member of the family has the same job and hours, but working less due to children at home/other caregiving responsibilities
- Another member of the family is working from home/remotely - required by employment/government mandate
- We have gone from 2 earners to 1
- We have gone from 2 earners to 0
- We have gone from 1 earner to 0
- We have significantly reduced income (cannot quantify with choices listed above).
- No economic impact
- I prefer not to answer.

Q92 Which of the following has four legs? This is a data quality check.

- Worm
- Dog
- Human
- Fish
